# Organoids in biliary research: insights into developmental signaling and applications in disease modeling

**DOI:** 10.3389/fcell.2025.1656019

**Published:** 2025-09-03

**Authors:** Boming Peng, Min Huang, Jianquan Zhang, Yang Xiang

**Affiliations:** ^1^ Department of Hepatobiliary Surgery, Haikou Affiliated Hospital of Central South University Xiangya School of Medicine, Haikou, China; ^2^ Department of Anesthesiology, Haikou Affiliated Hospital of Central South University Xiangya School of Medicine, Haikou, China; ^3^ Haikou Key Laboratory of Clinical Research and Transformation of Digestive Diseases, Haikou, China

**Keywords:** organoids, biliary diseases, signaling pathways, translational medicine, personalized medicine

## Abstract

The embryonic development of the biliary system is orchestrated by complex signaling pathways, including Notch, TGF-β, Wnt, and GFs, which regulate biliary stem/progenitor cell fate, polarity, and ductal morphogenesis. These pathways not only govern physiological development but are also deeply implicated in pathological processes such as inflammation, fibrosis, and carcinogenesis. However, conventional two-dimensional culture systems and animal models fall short in replicating the spatial structure, developmental dynamics, and human-specific molecular context of the biliary tract. As a result, bile duct organoids derived from various cellular sources have emerged as powerful *in vitro* platforms, capable of reconstructing key features of biliary architecture and function. Organoids not only respond to, but also enable the controlled manipulation of, developmental signaling pathways. These systems have revealed the dual roles of Wnt, Notch, TGF-β, and GFs in both tissue homeostasis and disease progression. Recent studies have successfully applied biliary organoid models to explore the mechanisms underlying primary sclerosing cholangitis, biliary atresia, cholangiocarcinoma, and gallbladder cancer, identifying critical signaling axes and regulatory networks. This review systematically reviews the roles of key signaling pathways in bile duct development and their regulatory mechanisms in organoid construction, with a particular focus on the applications of organoid models in elucidating signaling pathway abnormalities, uncovering disease mechanisms, and identifying potential therapeutic targets. It further provides a perspective on their prospects and challenges in the development of precision therapeutic strategies and clinical translation.

## 1 Introduction

The formation of the biliary system is a highly complex developmental process governed by the precise regulation of multiple signaling pathways, including Notch, TGF-β, Wnt, and GFs. These core regulatory networks exhibit spatiotemporally specific expression and orchestrate the coordinated processes of stem/progenitor cell lineage specification and tissue morphogenesis. Although the intrahepatic and extrahepatic bile ducts form a continuous anatomical network, they exhibit distinct developmental trajectories during embryogenesis, including derivation from different progenitor cell populations, varying degrees of dependence on developmental signaling pathways, and heterogeneity in stem cell niches (Lemaigre, 2020). These mechanisms not only underpin the proper formation of the biliary system but also provide critical developmental insights into a range of biliary diseases, such as biliary atresia, congenital bile duct dilation, and cholangiocarcinoma. Moreover, due to the complex anatomical structure of the biliary tree and the challenges in accessing tissue samples, the pathogenic mechanisms of many biliary disorders have long lacked systematic investigation. As an emerging three-dimensional (3D) *in vitro* culture technology, organoids are capable of recapitulating the physiological structure and function of biliary tissue and have become a valuable platform for dissecting signaling pathways involved in bile duct diseases.

Organoids are *in vitro* three-dimensional tissue structures that can be derived from (pluripotent) stem cells, progenitor cells, and/or differentiated cells. Through cell–cell and cell–matrix interactions, they undergo self-organization to recapitulate certain structures and functions of native tissues *in vitro* (Marsee et al., 2021). Compared with traditional two-dimensional cell culture and animal models, organoid models possess distinct advantages. First, organoid cultures exhibit patient specificity and can reproduce tissue-like structures and functions outside the body. Second, in contrast to two-dimensional cell lines, organoids not only preserve the polarity, stemness, and differentiation potential of cholangiocytes, but also allow the integration of gene editing, pharmacological interventions, and multi-omics technologies to dynamically track the activation, transduction, and regulation of disease-associated signaling pathways. Moreover, compared with animal models, organoid cultures are technically more accessible and therefore better suited for in-depth biological investigations (Zhao et al., 2022). Consequently, organoids have been widely applied in drug development, personalized companion diagnostics, and cell-based therapies. At present, biliary organoid models have been successfully established from normal tissues, induced pluripotent stem cells (iPSCs), and patient-derived diseased tissues. These models have enabled the identification of aberrant activation or functional remodeling of several key signaling pathways—including Notch, Wnt, TGF-β, PI3K/AKT, and Hippo—in diseases such as primary sclerosing cholangitis (PSC), biliary atresia (BA), cholangiocarcinoma (CCA), and gallbladder cancer (GBC) (Kosar, 2018; Meng et al., 2021; Yogo et al., 2023; Obata et al., 2023).

The successful construction of biliary organoids relies on the coordinated regulation of multiple developmental signaling pathways, including Wnt, the TGF-β superfamily, GFs, and Notch. These pathways are broadly involved in stem cell maintenance, self-renewal, lineage commitment, and the spatial organization of epithelial structures. The incorporation of signaling modulators such as Wnt3a, A83-01, GF, DAPT, Forskolin, and Y-27632 into organoid culture media enables the regulation of relevant signaling pathways, thereby facilitating partial functional and structural maturation of organoids. For example, Wnt3a plays a pivotal role in maintaining stemness and promoting proliferation through the activation of Wnt signaling (Calder et al., 2024); DAPT regulates the differentiation of hepatoblasts into cholangiocyte-like cells by modulating Notch signaling (Baulies et al., 2020); A83-01 participates in the spatial specification of the biliary lineage via regulation of TGF-β signaling (Li P. et al., 2024); while growth factors such as FGF and EGF function critically in organoid expansion and maturation (Palaria et al., 2018). Rational combination and modulation of these signaling factors form the foundation for the successful construction of organoid models. It is noteworthy that the “developmental maintenance-type” signaling pathways relied upon during organoid construction may, under pathological conditions, exhibit aberrant activation or dysregulated control, thereby transforming into key mechanisms driving disease processes. For instance, the Wnt pathway normally promotes stem cell expansion, but in cholangiocarcinoma it is closely associated with the maintenance of tumor stemness, uncontrolled proliferation, and therapeutic resistance (Xue et al., 2021); similarly, Notch signaling plays an essential role in promoting biliary development and differentiation, yet its mutation or loss of expression can lead to the bile duct paucity phenotype observed in patients with Alagille syndrome (Kohut et al., 2021). The shared nature of these signaling pathways serves as a bridge, not only providing new strategies for the precise modeling of organoid-based disease systems but also establishing the prerequisite conditions for in-depth investigation of pathogenic mechanisms.

Therefore, biliary organoids are transitioning from “structural reconstruction tools” to dual-purpose platforms for deciphering pathogenic signaling. This review focuses on biliary organoids and signaling pathways. It first provides an overview of the major signaling pathways involved in biliary development. It then focuses on the regulatory mechanisms of relevant signaling pathways during biliary organoid construction. Finally, under various disease contexts, it systematically reviews the applications and significance of biliary organoid models in elucidating abnormalities of key signaling pathways, exploring disease pathogenesis, and identifying potential therapeutic targets, and further discusses their prospects and challenges in the development of precision therapeutic strategies and clinical translation.

## 2 Biliary development

Bile duct development is a highly precise and dynamically regulated process involving the spatiotemporal expression of multiple signaling pathways, lineage decisions of stem/progenitor cells, and the coordinated remodeling of tissue architecture. Although intrahepatic and extrahepatic bile ducts form a continuous anatomical network, they display significant developmental heterogeneity during embryogenesis, particularly in terms of cellular origin, signaling pathway dependency, and stem cell niche composition. Specifically, intrahepatic bile ducts primarily originate from hepatoblasts, which form a monolayer of ductal plate structures around the portal vein region. These structures undergo remodeling under specific signaling cues to generate mature intrahepatic bile ducts. In contrast, extrahepatic bile ducts are derived from a group of pancreatobiliary precursors located in the ventral foregut endoderm, which co-express SOX17 and PDX1 and possess bipotential differentiation capacity toward biliary or pancreatic lineages. This fundamental difference in developmental origin indicates that although intra- and extrahepatic bile ducts share certain regulatory pathways, their developmental programs are not entirely identical (Si-Tayeb et al., 2010).

During lineage specification and ductal morphogenesis, intrahepatic and extrahepatic bile ducts share several conserved signaling pathways. Among them, the Notch pathway is one of the most critical regulators of bile duct development. Particularly in intrahepatic bile ducts, Notch2–Jagged1 (JAG1) signaling induces expression of the downstream transcription factor Hes1, thereby directing hepatoblasts to differentiate into BECs. Notch signaling not only initiates the formation of the ductal plate but also maintains biliary epithelial identity and inhibits transdifferentiation toward the hepatocytic lineage. The downstream effector SOX9, a key early marker of the biliary lineage, is highly expressed during ductal plate formation and serves as a crucial mediator of Notch function (Minnis-Lyons et al., 2021). This pathway plays a vital role in both the morphogenesis and functional maintenance of the biliary system. Dysfunction of the Notch pathway is closely associated with Alagille syndrome, which is characterized by bile duct paucity and ductal malformation phenotypes, further underscoring its indispensable role in bile duct development (Kakuda et al., 2020).

The TGF-β signaling pathway is one of the key regulatory mechanisms in bile duct formation. In the embryonic portal region, TGF-β forms a concentration gradient that promotes the differentiation of hepatoblasts located near the portal vein toward a biliary fate, and it regulates downstream transcriptional programs via SMAD2/3 proteins to precisely define the spatial boundaries of biliary differentiation (Gough et al., 2021; Schaub et al., 2018). The intensity of TGF-β signaling plays a decisive role in biliary lineage specification: high signaling strength induces loss of stem cell pluripotency and promotes differentiation into BECs, whereas insufficient signaling impairs bile duct development, leading to structural abnormalities in the biliary system (Antoniou et al., 2009). In addition, excessive activation of TGF-β can induce the transdifferentiation of hepatocytes into cholangiocyte-like cells, manifesting as a typical ductular reaction, and is closely associated with liver fibrosis and tumor progression. Furthermore, this pathway also contributes to bile duct lumen morphogenesis by promoting epithelial–mesenchymal transition (Rogler et al., 2009).

The Wnt/β-catenin signaling pathway exerts a complex and finely tuned regulatory role in bile duct development. On one hand, activation of this pathway promotes the expansion of hepatoblasts and favors hepatocytic lineage commitment; on the other hand, its moderate inhibition facilitates the maintenance of biliary fate. Studies have shown that during the early stages of ductal plate formation, Wnt signaling may be locally suppressed to allow Notch signaling to play a dominant role, though it still serves as an important regulator in maintaining the balance between hepatic and biliary differentiation (Martinez Lyons and Boulter, 2021). In the specific process of biliary differentiation, Wnt signaling is closely associated with liver lobule zonation and bile duct maturation. The ligand Wnt2b, secreted by the lateral plate mesoderm, binds to Frizzled family receptors and activates downstream transcriptional programs mediated by β-catenin (TIAN et al., 2022). Wnt signaling not only promotes early hepatoblast differentiation but also cooperates with HNF4α to regulate lobular zonation. Loss of β-catenin may lead to delayed and abnormal bile duct differentiation, ultimately resulting in ductal disorganization or even cyst formation (Wilson et al., 2020).

The GF signaling pathways—particularly FGF10—is thought to regulate epithelial polarity establishment, cellular migration, and proliferation, thereby contributing to the morphogenesis of ductal structures. In extrahepatic bile duct development, FGF10 is considered an important regulatory factor for directing pancreatobiliary precursors toward the biliary lineage (Hart et al., 2003).

It is important to note that although the aforementioned signaling pathways are active in both intra- and extrahepatic bile duct development, their degree of dependency, timing of action, and cellular responsiveness differ significantly. For instance, the formation of the extrahepatic bile duct highly depends in early stages on SOX17^+^/PDX1^+^ pancreatobiliary precursors, whose developmental mechanisms resemble pancreatic lineage specification. SOX17 plays a decisive role in the fate choice between biliary and pancreatic lineages; its loss leads to the formation of aberrant pancreatic-like tissue in the extrahepatic region. The expression of PDX1 further supports their bipotential identity, a pattern not dominant in intrahepatic bile duct development. By contrast, intrahepatic bile ducts undergo lineage selection and morphogenesis beginning with the ductal plate structure, under the regulation of Notch2–JAG1 and TGF-β signaling. Notch and SOX9 expression is spatially restricted to the periportal region, while Wnt/β-catenin signaling is more involved in stem cell expansion and lineage balance. In extrahepatic bile ducts, however, Wnt activity appears to be relatively limited and may not serve as a major regulatory input (Si-Tayeb et al., 2010).

At the level of stem cell niches, intra- and extrahepatic bile ducts also differ substantially. In extrahepatic ducts, abundant peribiliary glands serve as important reservoirs of stem/progenitor cells, expressing high levels of EpCAM, SOX17, and PDX1, and exhibiting strong bipotency toward biliary and pancreatic lineages. In contrast, intrahepatic bile duct–associated stem cells are primarily located in the canals of Hering and around the ductal plate, where they play key roles in hepatic-biliary lineage maintenance and liver regeneration (Si-Tayeb et al., 2010).

In summary, bile duct development is a multilayered biological process involving multiple signaling pathways, distinct developmental stages, and intricate regulatory networks. A deep understanding of these mechanisms not only sheds light on the etiology of biliary diseases but also provides a theoretical framework and potential therapeutic targets for the precise construction of biliary organoids and their application in regenerative medicine.

## 3 Signaling pathways involved in the construction of biliary organoids

In the construction of biliary organoids, researchers commonly introduce exogenous signaling regulators to simulate the dynamic signaling environment observed during normal biliary development, thereby driving the self-renewal, lineage differentiation, and 3D structuring of “seed cells,” ultimately achieving the precise establishment of organoids. In this process, signaling pathways such as Wnt, TGF-β, Notch, and various GFs play central roles. Corresponding signaling molecules or small-molecule modulators are widely applied in culture systems, including Wnt3a (to maintain stemness and proliferation), A83-01 (a TGF-β receptor inhibitor that prevents premature differentiation), EGF (to promote epithelial cell proliferation), and DAPT (a γ-secretase inhibitor that blocks Notch signaling to induce differentiation). The combinations and dosage regulation of these factors enable organoids *in vitro* to more faithfully recapitulate the signaling networks underlying biliary development and regeneration, providing a critical foundation for elucidating the mechanistic roles of diverse signaling pathways in organoid construction

### 3.1 Wnt signaling pathway

During the cultivation of biliary organoids, the Wnt/β-catenin signaling pathway plays a central regulatory role in maintaining cellular viability, self-renewal, and inducing differentiation. By artificially modulating activating and inhibitory factors within the pathway, it is possible to precisely regulate its activation and suppression processes, thereby ensuring the stable growth and functional maintenance of biliary organoids.

During the construction of biliary organoids, activation of the Wnt signaling pathway relies on exogenous ligands and their enhancers. Wnt3a is a canonical activator that can directly bind to Frizzled receptors, inducing the activation of Dishevelled and promoting the accumulation of β-catenin in the cytoplasm followed by its translocation into the nucleus. This process subsequently activates the expression of multiple target genes and plays a critical role in stem cell maintenance and organoid construction (De Lau et al., 2011). R-spondin 1, as a signal potentiator, binds to LGR5 receptors and inhibits Wnt negative regulators—specifically the E3 ubiquitin ligases RNF43/ZNRF3—thereby enhancing the stability and activity of Wnt ligands. This promotes the accumulation of β-catenin and its transcriptional activation, supporting the growth and self-renewal of biliary organoids (De Lau et al., 2011). Conversely, the activity of the Wnt pathway can be effectively downregulated by inhibitory factors. IWP-2, a commonly used Wnt inhibitor, targets the Wnt ligand-modifying enzyme Porcupine, blocking ligand secretion and maturation, thus suppressing pathway activation. This strategy is often employed to induce differentiation of biliary organoids in specific directions or to investigate Wnt-dependent biological processes (Bengoa-Vergniory et al., 2014). Therefore, dynamic modulation of Wnt signaling activity not only accommodates the developmental requirements of organoid cultures at different stages but also provides an experimental foundation and interventional strategy for dissecting the roles of Wnt signaling in organogenesis and disease mechanisms.

It is noteworthy that although canonical Wnt/β-catenin signaling is generally considered essential for maintaining stemness and proliferation in most organoid systems, some studies (Wilson et al., 2020) have suggested that its role in the cultivation of biliary epithelial cell (BEC) organoids may be limited. In contrast, non-canonical Wnt/PCP signaling pathways mediated by ligands such as Wnt7a, Wnt7b, and Wnt10a have demonstrated more prominent functions in bile duct regeneration and injury repair. This finding not only challenges the conventional view that canonical Wnt signaling is indispensable across all organoid systems, but also offers novel signaling perspectives for the construction and optimization of biliary organoids.

### 3.2 TGF-β superfamily signaling pathways

TGF-β, Activin, and bone morphogenetic proteins (BMPs) are all members of the TGF-β superfamily and exert their regulatory functions on cell differentiation, proliferation, and functional maintenance through a shared SMAD-dependent signaling pathway. These signaling cascades play essential roles in biliary tissue development, homeostasis, and disease pathogenesis. Imbalances in these pathways may lead to aberrant bile duct cell fate decisions, thereby affecting tissue equilibrium, organogenesis, and various pathological processes.

In biliary organoid culture systems, inhibition of TGF-β signaling is commonly employed to maintain the undifferentiated state of stem cells and prolong their proliferative capacity. A83-01, a selective inhibitor of the TGF-β type I receptor (ALK5), effectively blocks activation of the TGF-β pathway and is widely used in stem cell and organoid culture systems (Li Z. et al., 2024). In addition, Activin A, as an important member of the TGF-β superfamily, activates the SMAD2/3 signaling pathway by binding to ActRIIA/ActRIIB receptors and their downstream ALK4/ALK7 receptor kinases. This, in turn, regulates the expression of a series of genes associated with endodermal lineage specification, stem cell fate maintenance, and differentiation, while also playing a critical role in the differentiation of stem cells into hepatic, biliary, and pancreatic lineages (Teo et al., 2012).

Unlike TGF-β and Activin A, BMP signaling exerts its biological effects through SMAD1/5/8 and participates in processes such as cell differentiation, bile duct morphogenesis, and tissue repair (Palaria et al., 2018). During the formation of biliary organoids, BMP4 regulates cell fate via activation of the SMAD1/5/8 pathway, facilitating the formation of bile duct-like structures and playing an essential role in early germ layer induction and endodermal differentiation (Ogawa et al., 2021). Studies have also demonstrated that BMP4 can act synergistically with FGF signaling to regulate cholangiocyte differentiation, highlighting its multifaceted role in signaling network modulation (Teo et al., 2012). Similarly, BMP7 contributes to the proliferation and differentiation of BECs via the SMAD1/5/8 axis. Moreover, BMP7 has been shown to upregulate key components of the PI3K/AKT/mTOR pathway, thereby enhancing stem cell proliferation and regenerative capacity, which ultimately improves the survival and growth efficiency of organoids (Zhang et al., 2019). The activity of BMP signaling during cell fate regulation is also modulated by endogenous antagonists. Noggin, a well-characterized BMP inhibitor, directly binds to BMP2, BMP4, and BMP7, preventing their interaction with receptors and thus suppressing downstream signaling activation. This regulatory mechanism plays an important role in processes such as cell development and tissue homeostasis during biliary organoid construction (Groppe et al., 2002).

Members of the TGF-β superfamily not only exhibit signaling crosstalk but may also exert synergistic or antagonistic effects. Previous studies have reported that high concentrations of BMP4 can enhance Activin A-induced endoderm differentiation, thereby cooperatively promoting the formation of biliary or pancreatic progenitor cells (Teo et al., 2012). Therefore, during the cultivation of biliary organoids, the precise modulation of the dynamic balance among TGF-β, Activin, and BMP signaling is essential not only for maintaining cell fate determination and culture system stability, but also for providing key regulatory strategies and theoretical underpinnings for regenerative medicine, disease modeling, and drug screening.

### 3.3 Growth factor signaling pathways

GF signaling pathways constitute a critical network regulating cell proliferation, differentiation, migration, and survival, playing central roles in physiological and pathological processes such as tissue repair, organ regeneration, and tumorigenesis. Representative members of the GF family—FGF, EGF, and hepatocyte growth factor (HGF)—are widely applied in the construction of biliary organoids to modulate cell fate transitions and functional maintenance. These factors primarily exert precise regulation over cellular biological behavior through activation of classical signaling cascades such as MAPK/ERK, PI3K/AKT, and STAT3.

During the development and culture of biliary organoids, FGF signaling plays multiple roles: it not only induces the differentiation of hepatic progenitor cells (HPCs) toward the biliary lineage, but also participates in cell migration and proliferation. Studies have shown that the absence of FGF signaling can lead to impaired bile duct development and disrupted differentiation, thereby severely affecting the structural integrity and functional maturation of the biliary system (Palaria et al., 2018). At the molecular level, FGF4, FGF7, and FGF10 bind to members of the FGFR family, activating the MAPK/ERK and PI3K/AKT pathways to trigger a series of downstream cascades. Moreover, FGF signaling can act synergistically with the Wnt pathway to maintain cholangiocyte homeostasis and differentiation potential (Farooq et al., 2021). Notably, unlike FGF4 and FGF10, FGF7 may also directly activate the STAT3 signaling cascade to further regulate cellular behavior, providing a new perspective for understanding its function in biliary organoids (Dudka et al., 2010).

The EGF signaling pathway plays a critical role in the growth, differentiation, and survival of epithelial cells, and its function is equally essential in biliary organoids, which represent a 3D epithelial-derived model. By activating the MAPK/ERK, PI3K/AKT, and STAT3 pathways, EGF signaling promotes cell proliferation, survival, and functional maintenance. Similar to the FGF and HGF signaling pathways, EGF signaling also exhibits multiple pathway cross-talk; however, it demonstrates distinct specificity in certain transduction mechanisms. For example, EGF primarily activates PI3K through GAB1, whereas STAT3 activation depends on SRC family kinases, rather than the JAK or IRS1-dependent pathways commonly utilized by FGF/HGF signaling (Kiyatkin et al., 2006; Li et al., 2007). These features may provide a theoretical basis for the specificity of EGF in organoid development.

The HGF signaling pathway also depends on the MAPK/ERK, PI3K/AKT, and STAT3 cascades to regulate cholangiocyte proliferation, migration, and survival. Similar to FGF signaling, HGF activates PI3K via IRS1. However, its activation of STAT3 tends to rely more on the JAK pathway (Zhang et al., 2018), suggesting a degree of specificity and independence in its mechanism of action during biliary organoid formation.

In summary, although the FGF, EGF, and HGF signaling pathways differ in their downstream activation mechanisms, they collectively regulate the formation and functional maintenance of biliary organoids through shared signaling networks such as MAPK/ERK, PI3K/AKT, and STAT3. A deeper understanding of the interplay among these growth factor signaling pathways will aid in elucidating the molecular basis of biliary system development and provide theoretical support for the optimization of organoid culture systems and the precision treatment of biliary-related diseases.

### 3.4 Notch signaling pathway

During the construction and maintenance of biliary organoids, the Notch signaling pathway also plays a critical role. This pathway primarily functions through γ-secretase-mediated proteolytic cleavage of the Notch receptor, resulting in the release of the Notch intracellular domain (NICD). Upon entering the nucleus, NICD initiates the transcription of target genes, thereby regulating cell fate determination, the balance between proliferation and differentiation, and tissue homeostasis (Wu et al., 2019). By modulating the activity of the Notch pathway, it is possible to precisely guide lineage differentiation within biliary organoids, thereby improving their spatial organization and functional properties.

In experimental modulation, the γ-secretase inhibitor DAPT (N-[N-(3,5-Difluorophenacetyl)-L-alanyl]-S-phenylglycine t-butyl ester) is widely used to suppress activation of the Notch signaling pathway. By blocking NICD release, DAPT inhibits Notch-mediated signal transduction, thus enabling regulation of stem cell fate, differentiation status, and organoid morphology (Baulies et al., 2020). In biliary organoid culture systems, the moderate application of DAPT not only facilitates the balance between stem cell proliferation and differentiation but also provides a strategic tool for the precise regulation of biliary organoids in applications such as organ regeneration, disease modeling, and drug screening.

In conclusion, the Notch signaling pathway is involved in multiple key stages of bile duct development and exerts multilayered regulatory effects during the formation and functional maintenance of organoids. Its central role in cell fate determination makes it an important target for modulating organoid morphology and function, while also offering a theoretical foundation and experimental tool for understanding and intervening in biliary disease mechanisms.

### 3.5 Other signaling pathways

During the construction and maintenance of biliary organoids, in addition to the aforementioned microenvironmental signaling regulations that determine cell survival, proliferation, and functional maturation, Forskolin and Y-27632 are also widely applied in in vitro culture systems. These compounds exert critical auxiliary effects by activating the cAMP/PKA axis and inhibiting the RhoA/ROCK axis, respectively, thereby promoting functional enhancement and improving culture efficiency.

Forskolin is a classical activator of adenylyl cyclase that significantly elevates intracellular levels of cAMP, thereby activating the PKA signaling pathway. This pathway is broadly involved in various physiological processes, including ion transport, secretion regulation, maintenance of cell polarity, and metabolic modulation. In organoid systems, Forskolin has been shown to enhance epithelial secretory activity and lumen formation. Specifically, in biliary organoids, Forskolin promotes the expansion and morphological maturation of bile duct-like luminal structures, thereby improving their physiological functionality and overall growth status (Verplank et al., 2019).

**FIGURE 1:**
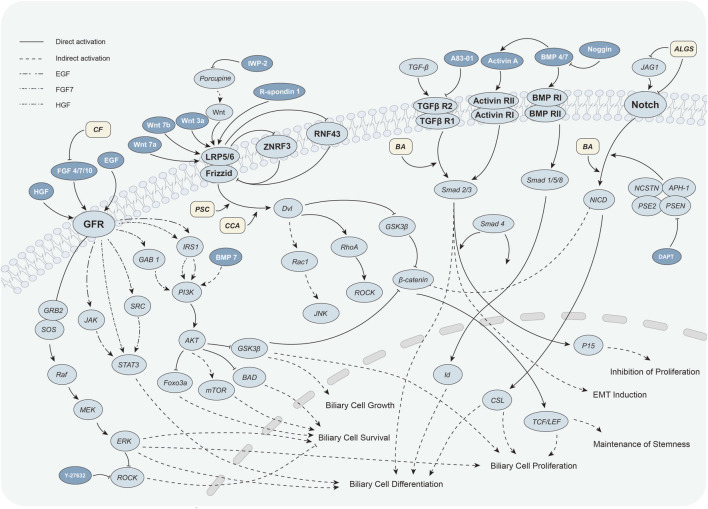
Key signaling molecules and pathways involved in biliary organoid construction and biliary disease mechanisms. In biliary organoid construction, the Wnt/β-catenin, Notch, TGF-β, and growth factor (GF) pathways play central roles. Critical downstream effectors (e.g., LGR5, SMAD2/3) as well as major activators (Wnt3a, EGF, HGF) and inhibitors (IWP-2, DAPT, Noggin) are indicated to highlight their regulatory roles in cholangiocyte differentiation, survival, and proliferation. In the disease context, aberrant signaling promotes disease progression. For instance, Notch–JAG1 mutations in Alagille syndrome (ALGS) impair cholangiocyte proliferation, while abnormal Wnt activation enhances cholangiocyte growth and survival. Arrows denote activation (→), blunt-ended lines indicate inhibition (⊥).

Y-27632 is a selective inhibitor of ROCK that mainly functions by blocking the RhoA/ROCK signaling pathway. It suppresses stress responses associated with cytoskeletal remodeling and significantly reduces apoptosis rates. During single-cell seeding, initial construction, and serial passaging of biliary organoids, Y-27632 has been shown to markedly enhance cell adhesion, survival efficiency, and colony-forming capacity, thereby supporting the maintenance of cellular homeostasis and improving culture success rates (Watanabe et al., 2007) (Figure 1).

## 4 Disease-associated signaling pathways in biliary disorders

The value of biliary organoids lies not only in their ability to recapitulate the structural and functional environment of the bile duct *in vitro*, but more importantly, in providing an ideal platform for elucidating the pathogenic mechanisms of biliary diseases, validating dysregulation of key signaling pathways, and exploring potential therapeutic strategies. Therefore, investigating aberrant regulation of signaling pathways in biliary diseases using organoid models has become a major focus in this field (Table 1).

**TABLE 1:** Research progress on key signaling pathways in biliary organoid disease models.

Biliary disease	Involved signaling pathways	Main findings	Research scope
PSC	PI3K/ERK, NF-κB	hP-MSCs upregulate TGR5 to activate PI3K/ERK and NF-κB signaling, thereby suppressing inflammation and fibrosis	Disease mechanism research
	Wnt	Wnt7A promotes hepatocyte transdifferentiation, Wnt7B promotes cholangiocyte proliferation, both synergistically improving cholestasis	Disease mechanism research
	FXR-SHP	Activation of FXR-SHP signaling reduces ROS, alleviates bile acid accumulation and inflammatory injury	Metabolic regulation and pharmacological intervention research
	PERK/CHOP	MSC-derived exosomes inhibit Th17 differentiation, alleviating ER stress and fibrosis via PERK/CHOP signaling	Metabolic regulation and pharmacological intervention research
BA	Notch	aHSCs activate Notch signaling, leading to abnormal proliferation of CK19+ cells and pathological duct formation	Disease mechanism research
	TGF-β	KPN infection activates IL-13/TGF-β1, driving biliary fibrosis	Disease mechanism research
	CaMKK2-AMPK	NT inhibits the CaMKK2-AMPK pathway, impairing bile duct regeneration	Metabolic and regenerative regulation mechanism research
	mTOR	Aβ and D-2-HG inhibit mTOR signaling, impairing energy metabolism and liver regeneration	Metabolic and regenerative regulation mechanism research
	CCL2-CCR2	TNFRSF12A drives inflammation via the CCL2-CCR2 axis	Metabolic and regenerative regulation mechanism research
	TWEAK/FN14	TWEAK/FN14 signaling accelerates HPC-mediated fibrosis; its inhibition attenuates pathological responses	Disease mechanism research
	Hippo-YAP1	Aberrant activation of the Hippo-YAP1 pathway induces oxidative stress, disrupting bile duct development	Metabolic and regenerative regulation mechanism research
CF	cAMP/ERK	AC5 activates the cAMP/ERK pathway to induce cyst-like proliferation; inhibitors suppress this growth	Disease mechanism research
	FXR	Gut microbiota dysbiosis suppresses the FXR-Fgf15 axis; exogenous bile acids can restore FXR activity	Disease mechanism research
ALGS	Notch	Jag1/Notch2 deficiency causes defects in bile duct branching; IGF1 can partially restore proliferation	Disease mechanism research
	PCP	Vangl2 knockout results in PCP pathway dysfunction, disrupting cell polarity and bile duct morphogenesis	Disease mechanism research
CCA	WNT	DRD1 inhibition upregulates WNT7B, promoting tumor proliferation	Disease mechanism and targeted intervention research
	PI3K/AKT	miR-451a inhibits the PI3K/AKT pathway, suppressing tumor growth and inducing apoptosis	Disease mechanism research
	YAP	LCK inhibitors suppress tumor growth significantly by inhibiting YAP activity	Disease mechanism and targeted intervention research
	NFκB	CircPCSK6-encoded protein inhibits the NF-κB pathway, thereby slowing ICC progression; NETs activate NF-κB signaling to promote CCA proliferation and metastasis	Disease mechanism and targeted intervention research
	Oleic acid–PPARγ–FABP4	The oleic acid–PPARγ–FABP4 loop promotes lymph node colonization and spread of CCA through fatty acid metabolic reprogramming	Disease mechanism research
	IL-17A	IL-17A enhances proliferation of PSC-CCA tumor cells	Disease mechanism research
	TNFSF9	HBV suppresses antitumor immune responses via TNFSF9 signaling	Disease mechanism and targeted intervention research
GBC	SEMA4A	Inhibition of SEMA4A signaling may prevent the progression of BilIN to adenocarcinoma	Disease mechanism research
	PI3K/HDAC	The dual PI3K/HDAC inhibitor CUDC-907 significantly suppresses tumor growth	Disease mechanism, drug screening, and targeted intervention research
	EREG/EGFR/mTORC1	ELF3 downregulates the EREG/EGFR/mTORC1 axis, thereby inhibiting GBC growth	Disease mechanism and targeted intervention research

### 4.1 Primary sclerosing cholangitis

PSC is considered an immune-mediated liver disease of multifactorial and polygenic origin, potentially involving cell-mediated hyperimmune responses that target BECs (Zecher et al., 2024). As a highly biomimetic 3D *in vitro* culture model, organoids can recapitulate the native tissue architecture and intercellular interactions, making them a powerful tool for investigating the pathogenic mechanisms and regulatory signaling pathways involved in PSC. By utilizing biliary organoids derived from PSC patients, researchers can reconstruct the pathophysiological features of bile ducts in a controllable *in vitro* setting. This enables in-depth analysis of key pathological processes, including bile stasis, chronic inflammation, and biliary fibrosis, and facilitates the identification of potential therapeutic targets, thereby providing an ideal platform for mechanistic exploration and precision treatment development.

During normal development of the biliary system, the PI3K/AKT and Wnt signaling pathways play essential roles in maintaining cholangiocyte survival, proliferation, and functional differentiation. The PI3K/AKT pathway supports long-term expansion and homeostasis in hepato-biliary organoid cultures by regulating cell cycle progression, anti-apoptotic mechanisms, and metabolic balance, and is one of the most commonly employed growth-promoting signals in organoid systems. Meanwhile, the Wnt pathway is pivotal for stem cell self-renewal, maintenance of biliary lineage identity, and spatial tissue organization, particularly in the formation of polarized structures and bile duct–like lumens in organoids. However, in disease models, these physiological signaling pathways often exhibit aberrant activation or dysregulation, becoming key drivers of disease progression. In studies of PSC-related injury mechanisms, Yao et al. (2023) demonstrated that human placenta-derived mesenchymal stem cells significantly upregulate the TGR5 receptor, subsequently activating the PI3K/ERK and NF-κB pathways. This activation suppresses the expression of pro-inflammatory and pro-fibrotic genes, thereby alleviating inflammatory responses and fibrotic deposition in PSC organoid models. Building on this, Kosar (2018) showed that Wnt7A signaling promotes transdifferentiation of hepatocytes into cholangiocyte-like cells, while Wnt7B enhances proliferation of BECs. Together, these pathways synergistically improve bile stasis phenotypes in PSC organoids, highlighting the critical role of Wnt signaling in bile duct regeneration and bile flow regulation.

In the context of metabolic regulation and therapeutic intervention, Yao et al. (2024) developed a reactive oxygen species (ROS)-responsive nanoparticle system to deliver obeticholic acid to PSC organoids. Activation of the FXR-SHP signaling axis was shown to reduce ROS levels, alleviate bile acid accumulation, and mitigate inflammatory injury in cholangiocytes. In another study, Chen et al. (2024) treated PSC organoids with hP-MSC–derived exosomes and found that they suppressed Th17 cell differentiation and alleviated endoplasmic reticulum (ER) stress–induced fibrosis by modulating the PERK/CHOP pathway. These findings suggest that mesenchymal stem cells and their derivatives may exert anti-fibrotic effects through both immune and stress-response modulation. Additionally, Stein et al. (2021) reported that IL-17A/F induces upregulation of PD-L1 in cholangiocytes, thereby inhibiting CD8^+^ T cell–mediated cytotoxicity and partially mitigating biliary inflammation. This finding suggests a dual role of the IL-17 cytokine family in PSC immune regulation, wherein it may amplify inflammation but also exert immunosuppressive effects under specific conditions.

In summary, PSC organoid models demonstrate remarkable biomimetic capabilities in recapitulating biliary epithelial injury, inflammation, and fibrotic remodeling. They offer new perspectives for dissecting key signaling pathways implicated in PSC pathogenesis. Existing studies have confirmed the pathogenic potential and therapeutic relevance of pathways such as PI3K/ERK and Wnt in PSC. These insights not only provide candidate strategies for targeted therapy but also lay a solid experimental foundation for personalized drug screening and precision medicine in the context of biliary disease.

### 4.2 Biliary atresia

BA is a severe neonatal cholangiopathy that typically manifests within the first few weeks after birth as persistent jaundice and pale stools. Although the etiology of BA remains unclear, its pathogenesis involves the dysregulation of multiple signaling pathways (Tam et al., 2024). In recent years, BA organoid models have emerged as critical tools for investigating signaling pathways associated with bile duct injury, bile stasis, and fibrosis.

During biliary development and organoid construction, the Notch and TGF-β signaling pathways play pivotal roles in cholangiocyte lineage specification and biliary regionalization, respectively. However, in disease states, these developmental pathways often become aberrantly activated and serve as major drivers of pathological bile duct remodeling. Brovold et al. (2021), using a 3D fibrotic microenvironment organoid model incorporating activated hepatic stellate cells (aHSCs), demonstrated that aHSCs induce pathological proliferation of CK19^+^ cholangiocyte-like cells via activation of the Notch signaling pathway, resulting in abnormal ductal structures. These findings highlight the central role of Notch signaling in pathologic ductal hyperplasia in BA, indicating a shift from its physiological role in lineage induction to a pathological driver of cellular overproliferation. In addition, Meng et al. (2021) reported that microbial infection may also drive biliary pathology through reprogramming developmental signaling pathways. In a BA organoid model, infection with *Klebsiella pneumoniae* significantly activated the IL-13/TGF-β1 axis, thereby inducing a fibrotic response in cholangiocytes. This suggests that, under infectious conditions, the TGF-β pathway may be reprogrammed into a pro-fibrotic signaling axis, promoting pathological remodeling of the biliary microenvironment and contributing to BA progression.

Studies exploring metabolic and regenerative regulation in BA have also identified key signaling disruptions. Zhao et al. (2024) found elevated levels of neurotensin in BA patients, which suppress cholangiocyte proliferation via inhibition of the CaMKK2-AMPK signaling pathway, suggesting NT may impair bile duct regenerative capacity. Tian et al. (2022a); Tian et al. (2022b) further demonstrated that β-amyloid and D-2-hydroxyglutarate inhibit mTOR signaling, resulting in impaired energy metabolism and hepatobiliary regeneration in BA organoids, thereby emphasizing the critical role of metabolic dysregulation in disease progression. Additionally, single-cell transcriptomic analysis of BA organoids by Xiao et al. (2024) revealed that TNFRSF12A drives pathological inflammatory responses in cholangiocytes via the CCL2-CCR2 axis, indicating a potential anti-inflammatory therapeutic target.

Further studies have implicated additional signaling axes in the regulation of fibrosis in BA. Short et al. (2023) identified the TWEAK/FN14 pathway as a promoter of fibrosis in Prominin-1^+^ HPCs, and inhibition of this pathway effectively reduced HPC-mediated pathological responses. Xie et al. (2024) demonstrated that aberrant activation of the Hippo-YAP1 pathway induces oxidative stress and suppresses the antioxidant factor PRDX1, impairing cholangiocyte development and positioning YAP1 as a potential regulatory target for bile duct regeneration.

Collectively, BA organoid models have been widely applied to dissect the roles of key signaling pathways—including Notch and TGF-β—in bile duct injury, bile stasis, and fibrotic remodeling, which are central to BA pathophysiology. These studies not only deepen our understanding of BA pathogenesis but also provide experimental and theoretical foundations for the development of targeted therapeutic strategies. Moving forward, precise modulation of these signaling pathways holds promise for improving BA organoid model fidelity and advancing their translational potential in mechanistic studies and clinical applications.

### 4.3 Cystic fibrosis

Organoid models have demonstrated great potential in elucidating the pathogenic mechanisms of cystic fibrosis (CF), particularly in the study of disease-related signaling pathways. CF organoid systems not only enable functional modeling of mutant CFTR (cystic fibrosis transmembrane conductance regulator) dysfunction but also serve as essential experimental platforms for investigating the mechanisms of CF-related complications in organs such as the biliary tract and for exploring targeted interventions. Bijvelds et al. (2022) established monolayer models using intestinal and biliary organoids derived from CF patients to systematically analyze the impact of CFTR dysfunction on ion transport. Their results showed that the classical CF mutation Phe508del causes impaired Cl^−^ and HCO_3_^−^ transport, disrupting normal secretory functions in both the intestine and biliary epithelium. The triple combination therapy of elexacaftor–ivacaftor–tezacaftor (ELX/IVA/TEZ) effectively restored the function of the mutant CFTR channel, significantly promoting ion secretion. This supports its clinical potential in CF treatment, particularly for personalized therapeutic strategies in patients with biliary involvement.

In the context of CF-related polycystic liver disease (PLD), Spirli et al. (2017) utilized a Pkd2-knockout mouse model to generate biliary organoids and demonstrated that adenylyl cyclase 5 (AC5) promotes cyst-like structure overgrowth through activation of the cAMP/ERK signaling pathway. This pathway was found to be regulated by ER calcium homeostasis [(Ca^2+^)ER]. Moreover, they showed that inhibition of AC5 markedly suppressed cyst growth at the organoid level, suggesting its potential as a therapeutic target for PLD, especially for autosomal dominant polycystic liver disease. Additionally, Ikpa et al. (2020) used CF organoid models to further uncover the bile acid feedback dysregulation within the gut–liver axis in CF. They found that FXR signaling activity was significantly diminished in CF mice, primarily due to suppressed downstream Fgf15 expression caused by gut microbiota dysbiosis. This finding was validated in CF organoid models, where exogenous bile acid stimulation successfully restored FXR activity, confirming the pivotal role of the “gut microbiota–bile acid–FXR” axis in CF-associated hepatobiliary complications. These results also provide theoretical and translational support for microbiota-targeted therapeutic strategies.

In conclusion, CF organoid models offer robust capabilities for recapitulating CFTR channel dysfunction, signaling pathway dysregulation, and organ-specific pathologies. Relevant studies have elucidated the roles of key pathways, including CFTR ion transport, cAMP/ERK, and FXR, in CF pathogenesis and validated the therapeutic efficacy of targeted interventions. Collectively, these findings not only advance our molecular understanding of CF but also provide a strong experimental foundation and translational prospects for the development of personalized treatments, targeted therapeutics, and strategies to mitigate CF-related complications.

### 4.4 Alagille syndrome

Alagille syndrome (ALGS) is a hereditary hepatobiliary disorder characterized by bile duct paucity, intrahepatic cholestasis, and multisystem abnormalities resulting from impaired bile duct development (Sutton et al., 2024). In recent years, organoid models have demonstrated distinct advantages in elucidating the pathogenesis of ALGS, particularly in identifying key signaling pathways underlying bile duct developmental defects and in screening potential therapeutic targets.

Among known mechanisms, the Notch signaling pathway is widely regarded as the central pathogenic driver of ALGS. More than 94% of patients who meet clinical diagnostic criteria for ALGS harbor mutations in JAG 1, which encodes a conserved ligand of the Notch pathway; a minority (2%–3%) possess mutations in NOTCH2, encoding one of the four Notch receptors. The mutation spectra of both JAG1 and NOTCH2 have been well characterized (Gilbert et al., 2019). Roos et al. (2022), using human-derived branching biliary organoids, demonstrated that the JAG1/NOTCH2 signaling axis plays a crucial regulatory role in bile duct branching morphogenesis. Jag1 mutations impair Notch2 signaling, resulting in structural bile duct abnormalities that recapitulate ALGS bile duct phenotypes. Similarly, Iqbal et al. (2024) established organoids derived from the hilar (hICOs) and peripheral (pICOs) regions of Jag1 (Ndr/Ndr) mutant mice to investigate regional differences in Notch signaling. Their study revealed that hICOs, under Notch-deficient conditions, lost dependence on IGF1, while exogenous supplementation of IGF1 partially restored the proliferative capacity of pICOs, suggesting that IGF1 may serve as an auxiliary therapeutic strategy to promote bile duct regeneration in ALGS. In addition, studies by Andersson et al. (2018) and Fabris et al. (2007) indicated that Notch signaling deficiency not only suppresses normal proliferation of cholangiocytes but also leads to the loss of function in reactive ductular cells and abnormal accumulation of hepatobiliary cells, thereby impairing the liver’s regenerative response and exacerbating tissue damage.

Beyond the Notch signaling pathway, organoid models have also been employed to investigate additional signaling mechanisms implicated in bile duct developmental defects in ALGS, broadening our understanding of the disease’s multifactorial etiology. In the context of bile duct morphogenesis, Raab et al. (2024) developed a Vangl2 knockout organoid model and discovered that disruption of the PCP signaling pathway impairs the polarity alignment and intercellular connectivity among cholangiocytes, ultimately resulting in failure of lumen formation. This finding highlights the critical role of PCP signaling in spatial organization and morphogenesis during late-stage bile duct development.

In summary, ALGS organoid models have enabled the dissection of multiple key signaling pathways involved in abnormal bile duct development, including JAG1/NOTCH2 and PCP pathways. Region-specific defects in Notch signaling predominantly affect cholangiocyte proliferation and lineage maintenance, while PCP signaling regulates the spatial and morphological establishment of bile duct lumens. These studies have not only deepened our understanding of ALGS pathogenesis but also provided important experimental evidence and theoretical rationale for the development of future targeted therapies.

### 4.5 Cholangiocarcinoma

CCA is a highly heterogeneous and aggressive malignancy with extremely poor prognosis. Based on anatomical location, it is classified into intrahepatic (iCCA), perihilar (pCCA), and distal (dCCA) subtypes. The pathogenesis of CCA is complex, involving the aberrant activation of multiple signaling pathways, which significantly limits the efficacy of current therapeutic strategies (Zaki et al., 2023). In recent years, the application of organoid technology—a highly biomimetic 3D culture model—has emerged as a powerful platform for faithfully recapitulating the genetic background, tissue architecture, and microenvironmental features of tumors *in vitro*. This has enabled the exploration of key oncogenic signaling pathways and the development of individualized therapeutic strategies with unprecedented precision.

Organoid models have shown distinct advantages in elucidating the pathogenic mechanisms of CCA. Yogo et al. (2023) demonstrated that the WNT7B-mediated Wnt signaling pathway was upregulated upon inhibition of dopamine D1 receptor signaling, thereby promoting CCA cell proliferation, suggesting that targeting the Wnt pathway may offer therapeutic potential. Obata et al. (2023) found that miR-451a suppressed tumor cell growth and induced apoptosis by negatively regulating the PI3K/AKT pathway, highlighting it as a candidate therapeutic factor in PI3K/AKT-dependent CCA. In molecular subtyping studies, Lee et al. (2023) revealed through multi-omics analysis that the large duct subtype of iCCA was enriched in KRAS signaling, indicating the potential of this pathway as a subtype-specific therapeutic target. Conboy et al. (2023) further validated in patient-derived organoids (PDOs) that the LCK inhibitor NTRC 0652-0 significantly suppressed tumor growth via inhibition of YAP activity, underscoring YAP as a viable therapeutic target.

CCA organoids also offer valuable insights into the regulation of tumor proliferation and signaling dynamics. Guan et al. (2025) reported that the 167-aa protein encoded by CircPCSK6 inhibited ICC progression via suppression of the NF-κB pathway and enhanced sensitivity to gemcitabine, suggesting its utility in chemopotentiation and targeted therapy. Zhou et al. (2024) found that exosomes derived from circulating tumor cells could transmit the long non-coding RNA TTN-AS1, promoting CCA cell proliferation and migration, positioning it as a potential diagnostic and therapeutic target. In the context of invasion and distal metastasis, Zhang H. et al. (2024) used lymph node microenvironment organoids to show that the oleic acid–PPARγ–FABP4 axis drives CCA colonization and dissemination through fatty acid metabolic reprogramming. In another study, Zhang C. et al. (2024) revealed that neutrophil extracellular traps promote CCA proliferation, migration, and angiogenesis by activating the ITGAV/NF-κB pathway, highlighting their pathogenic role within the tumor immune microenvironment.

In reconstructing the tumor immune microenvironment, organoids present unique advantages. Lieshout et al. (2023) showed that IL-17A promotes tumor cell proliferation in PSC-associated CCA organoids, suggesting the IL-17A axis as a potential immune therapeutic target for this disease subtype. Similarly, Li Z. et al. (2024) found that hepatitis B virus infection suppresses antitumor immunity through the TNFSF9 pathway, and inhibition of TNFSF9 enhances treatment sensitivity, unveiling a novel mechanism of immune modulation in virus-associated iCCA.

In conclusion, CCA organoid models offer a robust experimental platform for investigating the roles of multiple key signaling pathways in CCA pathogenesis. These studies have deepened our understanding of CCA heterogeneity, metastatic mechanisms, and immune evasion, while also laying a solid theoretical and experimental foundation for the development of individualized interventions such as targeted therapy, metabolic reprogramming, non-coding RNA regulation, and immunotherapy.

### 4.6 Gallbladder cancer

GBC is a highly lethal malignancy of the biliary system, characterized by insidious onset, strong invasiveness, and frequent diagnosis at an advanced stage. Its pathogenesis is complex, involving aberrant activation of multiple signaling pathways, which severely limits the efficacy of current therapeutic strategies (Siddiqui et al., 2025). Organoid models, owing to their unique capability of recapitulating organ-level architecture and functionality, have shown significant promise in elucidating the molecular mechanisms of GBC and exploring its targeted therapeutic potential.

In the study of GBC pathogenesis, organoid technology integrated with multi-omics approaches has provided new insights into key regulatory nodes of disease progression. Pirenne et al. (2024), through spatial transcriptomic analysis, found that suppression of SEMA4A signaling plays a critical regulatory role in the progression from biliary intraepithelial neoplasia to adenocarcinoma, suggesting its potential as a biomarker for malignant transformation in precancerous lesions. In addition, Oda et al. (2024), using GBC organoids derived from Kras/Trp53 double-mutant mice and human GBC cell lines, identified that miR-34a-5p suppresses GBC cell proliferation and cell cycle progression by downregulating CDK6 expression, suggesting its potential as a nucleic acid-based therapeutic target.

In the realm of molecular targeted therapy for GBC, Yuan et al. (2022) employed PDOs for drug screening and discovered that the dual PI3K/HDAC inhibitor CUDC-907 significantly inhibited tumor growth, underscoring the potential of PDOs in individualized therapy screening. Furthermore, Nakamura et al. (2023) reported that the transcription factor ELF3 suppresses GBC cell proliferation by downregulating the EREG/EGFR/mTORC1 signaling axis, suggesting that EGFR/mTORC1 inhibitors may offer enhanced therapeutic efficacy in GBC patients with low ELF3 expression.

In summary, GBC organoid models have proven instrumental in uncovering the regulatory roles of key oncogenic pathways such as PI3K/AKT, EGFR/mTORC1, and CDK6 in GBC development and progression. They also demonstrate considerable potential in studying precancerous lesion transformation, targeted therapy screening, miRNA-based intervention, and the mechanistic roles of infectious factors. These findings provide a solid theoretical foundation and experimental framework for advancing precision medicine, early disease detection, and personalized intervention strategies for GBC.

## 5 Conclusions and perspectives

Organoid technology has provided a novel experimental platform for studying signaling pathways involved in biliary physiology and pathophysiology. This approach offers significant advantages in recapitulating biliary tissue architecture, preserving patient-specific genetic backgrounds, and supporting high-throughput screening applications. Using organoid models, researchers have identified reprogramming of key pathways—such as Notch, Wnt, TGF-β, PI3K/AKT, and Hippo—during the pathogenesis of various biliary diseases, and have preliminarily explored the feasibility of targeted interventions.

However, current biliary organoid models still exhibit significant limitations in faithfully recapitulating the *in vivo* physiological and pathological microenvironment. First, the absence of immune cells and stromal components hampers their ability to model immune-mediated injury responses, fibrogenesis, and tumor–microenvironment interactions (Wang et al., 2025). Second, insufficient reproduction of the biliary mechanical milieu in vivo—such as shear stress, hydrodynamic pressure, and matrix stiffness—may impair cholangiocyte polarity, differentiation, and signal regulation (Fiorotto et al., 2023). In addition, during long-term culture, organoids are prone to phenotypic drift and batch-to-batch variability, which compromises their stability in chronic biliary disease research and reproducibility in experimental studies (Jensen and Little, 2023). Moreover, current biliary organoid culture systems largely employ uniform conditions and lack the capacity to personalize the microenvironment according to specific disease states, thereby limiting their potential for precision disease modeling and individualized therapeutic strategy development.

Beyond these surface-level limitations, deeper scientific challenges remain to be addressed in order to advance organoids toward becoming truly biomimetic models of biliary diseases. First, biliary development and disease are not driven by single pathways but instead emerge from dynamic crosstalk among multiple signaling networks—for example, the antagonistic interaction between Wnt and Notch during cholangiocyte lineage differentiation (Siebel and Lendahl, 2017), or the potential synergistic effects of TGF-β and Hippo signaling in fibrosis and tumorigenesis (Zhang et al., 2025). How to decipher and reconstruct such signaling interactions within organoid systems is a critical step to enhancing their biomimicry. Second, the biliary system itself displays significant spatial heterogeneity: intrahepatic and extrahepatic bile ducts differ in stem cell niches, signaling dependencies, and disease susceptibility, thus necessitating the development of organoid models that reflect distinct anatomical subregions. Third, genetic and phenotypic drift during long-term organoid culture may arise from selective pressure or clonal dominance induced by sustained pathway activation. Addressing this challenge requires integrating single-cell multi-omics with gene editing approaches to uncover the underlying mechanisms and designing dynamic culture strategies to improve stability (Thalheim et al., 2021; Zeng et al., 2024). Finally, on the translational front, achieving standardization and large-scale production of organoids while incorporating patient-specific genetic backgrounds remains a major bottleneck.

Looking forward, the primary tasks for advancing biliary organoid technology should focus on immune microenvironment reconstruction, precise disease stratification, and integration into clinical companion diagnostics, thereby unlocking their full potential in disease modeling, target discovery, and personalized therapeutics. Progress may be pursued along several directions: immune and stromal integration, mechanical microenvironment simulation, optimization of long-term culture stability, disease-specific microenvironment customization, and advancement toward high-throughput and precision applications. For immune and stromal integration, co-culture systems or compartmentalized microfluidic platforms incorporating Kupffer cells, fibroblasts, and immune cell subsets may recapitulate the dynamic epithelial–stromal–immune interactions. For mechanical microenvironment simulation, organ-on-chip systems and dynamic bioreactors can be employed to apply controlled shear stress, cyclic strain, and tunable matrix stiffness to reconstruct the biomechanical features of the native bile duct. For example, Carolina et al. (2024) combined microfluidic organ-on-chip platforms with human iPSC-derived liver organoids to generate vascularized constructs containing functional bile ducts, demonstrating the potential to model congenital biliary malformations. For long-term culture stability, optimization of medium composition, matrix formulation, and passaging strategies—together with real-time multi-omics monitoring—may reduce phenotypic drift and inter-batch variability. For disease-specific microenvironment customization, growth factor combinations, matrix composition, and metabolic conditions can be adjusted according to distinct pathological contexts, such as cholestatic, inflammatory, or neoplastic diseases, to better simulate disease-specific signaling networks. Finally, for high-throughput and precision applications, integration of organoid models with CRISPR-based gene editing, 3D bioprinting, multiplex imaging, and artificial intelligence-assisted data analysis could accelerate drug screening, target validation, and individualized therapeutic development.

Through these strategies, cholangiocyte organoids are expected to evolve from generalized culture systems into highly biomimetic, disease-specific research models, thereby enabling broader and more precise applications in disease modeling, target discovery, drug evaluation, and clinical translation.
